# Section Work in the U.S.V.M.A.

**Published:** 1897-04

**Authors:** W. H. Dalrymple

**Affiliations:** New Orleans, La.


### SECTION WORK IN THE U.S.V.M.A.

EDITORS JOURNAL COMPARATIVE MEDICINE, ETC.

DEAR SIRS: Our veterinary journals have recently contained articles with reference to the desirability of devoting a part of the time of the annual meeting of the U. S. V. M. A. to section work. I think there can hardly be two opinions as to the good results that would accrue from the giving up of a portion of the time of the convention to the consideration of subjects having a more practical bearing on the everyday work of the general practitioner. It seems to me that by so doing we would popularize our meetings and render them more attractive to the great bulk of the profession. The man who is making his living by general practice is more apt to be induced to become an active member of our national body if he finds that by attending the meetings he will receive information and instruction which are calculated to benefit him in his daily work. It matters not at what point our meetings may be held, there are members who have to travel long distances to be in attendance; if, however, we can offer attractions to the general practitioner, and make him feel that it will pay him to become "one of us," I think we will have accomplished a great deal; because, after all, it is the profession as a whole that we have to look to for the encouragement and growth of our organization. By dividing up the time of our meeting, then, so as to cater to the wants of the various branches of the profession, we will not only add to the general interest taken in the Association, but largely augment our roll of membership.

If in order, I should like to express the hope that our Executive Committee is bearing Nashville, Tenn., in mind for the annual meeting of 1897. The profession in the South not only desires, but *requires* the aid and stimulus of our National Association; and as the profession south of Mason and Dixon's line has never been favored with a visit from that body, I sincerely trust the Executive Committee will appreciate our necessities and realize the benefits to the profession in the southern part of the Union that would result from such a meeting.

W. H. DALRYMPLE, M.R.C.V.S.

NEW ORLEANS, LA.

